# The Future of St. Bartholomew's Hospital

**Published:** 1903-11-28

**Authors:** 


### HOSPITAL ADMINISTRATION.

### CONSTRUCTION AND ECONOMICS.

### THE FUTURE OF ST. BARTHOLOMEW'S HOSPITAL.

#### THE SUPPRESSED REPORT OF THE MEDICAL COUNCIL.

The suppressed report of the Medical Council and the opinions which they placed before the Lord Mayor's Committee have now come into our hands.

It will be seen that the Medical Council are unanimous in supporting the views which have been expressed in favour of an increase in the site, and for the provision of a modern hospital, including new wards, as advocated by Sir Henry Burdett and Mr. Andrew Motion. All impartial people who read the statements made by the Medical Council and staff must agree that as the opinions of these experts, although submitted to the Mansion House Committee, have been practically ignored by the Governors, the latter are not justified in appealing to the public for support for their present proposals. It follows that if the Governors persist in doing so they are not likely to raise the money which they require to enable them to carry out their scheme of patch-work and tinker.

The Medical Council supplied the Lord Mayor's Committee in full, in their report which is dated February 1903, with the communications they have made to the treasurer, almoners, and Governors upon the whole question of the purchase of land from Christ's Hospital, and the buildings which might be erected thereon. They show that for nearly three and a half years they have been in communication with the treasurer as to the defective state of the hospital, that they have urged their views by deputation and reports dated November 2nd, 1899; August 2nd, 1901; October 22nd, 1901; November 4th, 1901; December 11th, 1901; and June 26, 1902. We have not space to summarise all these various documents to-day, nor is it necessary, seeing that they resulted in a modification of the original plan submitted by the architects, and that the criticisms of the staff upon the third plan prepared by Mr. I'Anson in conjunction with Mr. Rowland Plumbe, make it sufficiently clear what the views of the medical staff are at the present time. We have made arrangements, however, by which anyone who is interested may obtain a copy of the full report of the medical staff from The Scientific Press, 28 and 29 Southampton Street, Strand, W.C.

It is, perhaps, material to quote the following paragraph from the report of the Governors, dated August, 1901:—

"On November 22nd, 1899, a deputation from the Medical Council urged upon the Treasurer and Almoners our conviction of the necessity for the acquisition of the whole of the site of Christ's Hospital. They stated our belief that the portion of the site asked for—about 1½ acre—would be quite inadequate, and that if this opportunity of gaining the whole site were lost, the proper growth of St. Bartholomew's would be hampered for all time."

It is, perhaps, material to quote the following paragraphs which appear on page 8 of the Report of the Medical Council, under the heading of "Conclusions":—

"In drawing up this report the Medical Council have been throughout influenced by the feeling that the Governors of St. Bartholomew's Hospital and the public they represent will demand that, if large sums of money are spent on new buildings, these latter must certainly approximate to the requirements of modern sanitation and hospital construction, and must be prepared to stand the test of comparison with other recently erected hospitals. It is generally conceded by all recognised authorities, *vide* Sir Douglas Galpin and Sir Henry Burdett, that a minimum of 60 patients per acre of site is a standard that should be obtained if possible, and the present building of St. Thomas's Hospital may be cited as an example of what has been done in recent years in London, where 500 beds, with a medical school and nurses' home have been provided for on about eight acres of ground."

The Medical Council quote the following from the architects' report, which is very instructive as an indication of the origin of the present deadlock:—"We have taken it as absolutely settled that the hospital cannot acquire any more land adjoining its own site beyond that which has been arranged for. We further have not considered the question of any future rebuilding of the hospital; and, having regard to the suggestions of the House Committee, we have not taken into consideration the rebuilding of the hospital as a whole." The Medical Council record their "appreciation of the endeavours which the architects have made to meet their views." They then make some practical comments on the details of the plan for which we have no space. These comments are not so material to the issues at stake as their consideration of the general plan as a whole. This latter we therefore give verbatim.

#### The Plan as a Whole.

In their last report to the treasurer and almoners, dated June 26th, 1902, the Council recorded their unchanged conviction that the present site, including the area recently purchased, is insufficient for the erection of the buildings so urgently required now, and that it affords no provision whatever for the inevitable expansion of the hospital in times to come.

"Nothing has happened since then to alter the Council's opinion, and since the Governors have now decided—

(*a*) That the hospital cannot afford to buy more land, though such a step is confessedly desirable, and

(*b*) That an appeal must in any case be made to the public for funds,

They would respectfully urge that in appealing to the public the Governors should bear in mind the necessity of—

(1) Maintaining the income of the hospital;
(2) Erecting new buildings; and
(3) Obtaining more land.

The question now is whether to obtain such an area as shall suffice for the proper and healthy accommodation of the various departments of the hospital, which have always grown, and must always continue to grow, as medicine advances, or on the other hand to remain content with a site which is even now too small, and which renders any further improvement impossible.

The Council regret that their suggestion 'That it is highly desirable that there should be in existence some plan dealing with the whole site at present available quite irrespective of any existing buildings,' should not have been considered by the architects.

The Council would again respectfully urge upon the hospital authorities that it is of the greatest importance not to be content with the very least that the pressing needs of the moment require, but to take all steps, and to try all available means, to add to the area now at their disposal. They cannot but think that if the public were in full possession of the facts they would increase their subscriptions as they saw the increased need for them.

Our treasurer has often publicly deplored the common delusion that the hospital is too rich to need support.

Little has yet been done to dispel this illusion. Were it frankly stated in the appeal to the public that the Governors consider it of the first importance to the future of the hospital that more land should be obtained than our unaided resources can afford to buy, the Council firmly believe that the contributions would be increased."

*October*, 1901.

The Medical Councils Representations to the Lord Mayor's Committee.

The Medical Council desire to point out to the Lord Mayor's Committee, that whilst they have cordially approved of most of the proposed new buildings themselves, they have alway expressed the opinion that the site dealt with is, as a whole, insufficient. They have constantly kept in view the future rebuilding and expansion of the hospital, and it was with this object that in their report upon plan No. 2 they suggested that "there should be in existence some plan dealing with the whole site at present available, quite irrespective of any existing buildings."

In the reports hitherto quoted, the Council have dealt almost entirely with the new buildings proposed to be erected, and with the insufficiency of the proposed area.

The present ward blocks also require consideration. Any scheme of building must have regard to the future, because the time is probably not far distant when all the existing ward blocks will have to be replaced by others more in accord with modern medical requirements. They do not forget the efforts which the Governors have made to improve the existing wards in many ways; but the final limit to further improvement of the present buildings must come, even if it have not come already. The modern treatment of disease requires a much more liberal allowance of light, air, and space, in proportion to the number of patients, than was considered to be necessary when the present hospital was so ably planned 160 years ago. It has also been found to be of great advantage to the patients that isolation rooms, day rooms, and other adjuncts should be provided. The modern "ward unit" cannot be evolved from the existing ward buildings.

As a result of the foregoing considerations it becomes evident that the present area of six-and-a-half acres is insufficient for the erection of the whole of the buildings required by St. Bartholomew's Hospital. To cope with this difficulty several alternative plans may be suggested.

1. The acquisition of more adjoining land.
2. The erection of the nurses' Home (which occupies much of the site), as well as of the college, elsewhere.
3. The diminution of the number of beds.
4. The removal of the hospital to another site.

Of these alternatives the Medical Council are strongly in favour of the first, which would enable the hospital to be retained in its present position.

As regards the second alternative—namely, the erection of the nurses' home outside the hospital area, as has been done by other London hospitals—the Medical Council believe that although this would not be so satisfactory as obtaining more land, it would nevertheless permit of the retention of the hospital on its present site, It is manifest that it would be desirable to build a nurses' home at the hospital itself if enough land is obtainable, but if it be impossible to build it without seriously diminishing the number of beds, then there is no other proposed new building which could be placed elsewhere with so great advantage, and would at the same time supply more room by its removal than would this. For it must be noted that, as at present designed, the nurses' home presents a longer frontage in its three blocks than do the three ward blocks for about 600 patients, which will constitute nearly the whole in-patient department, and may fairly be considered the chief portion of the hospital, to which all other departments should be subservient.

The third alternative of diminishing the number of beds would be the ultimate and necessary consequence of proceeding with the plan now before the Governors (Plan 3). For there would be a loss of from 100 to 200 beds, if, after the proposed new buildings were erected, the rebuilding of the wards were undertaken on the same site as that they now occupy. As already mentioned, the modern treatment of disease requires a much more liberal allowance of light, air, and space per patient than was considered necessary when the hospital was built. Such a diminution of the number of beds would probably seem as objectionable to the Governors as it does to the Medical Staff.

The fourth alternative of a new site is one the Medical Council can only regard as a last resort; and it may be pointed out that whatever advantages may be claimed for other sites, the gravel soil, the excellent drainage, and the open air space of West Smithfield, combine to make the present site one which is in many respects quite ideal The situation also is admirable and most convenient for patients; indeed, far more so than any other place could be. For St. Bartholomew's is not only the hospital for the City, with its immense day population, but is also the only large hospital for the adjacent districts of Holborn, Clerkenwell, and Finsbury, and serves in addition the whole of the North and the North-East of London, including parts of Hoxton and Hackney, as well as the more remote districts, Islington, Dalston, Pentonville, Walthamstow, and Leytonstone. It is further most accessible, being placed very near the stations of the Central London, Metropolitan, and District Railways, whilst it is also close to many large railway termini and to the numerous omnibus and tram lines which converge towards the City.

This accessibility is of importance, for it is altogether too narrow a view to take, that a hospital should be limited in its work to the district in which it is placed, for St. Bartholomew's Hospital is free without payment or letter of recommendation to all poor people, and its reputation is so widely spread that it necessarily draws patients, not only from all parts of London, but from much further afield. It may, with truth, be said that there is probably no part of the United Kingdom or Colonies which has not sent patients to this charity. The benefits of St. Bartholomew's Hospital are indeed, in another sense, even yet more widely diffused, for the medical men who have for so many years issued from its school in a constant stream, have extended the fame of their hospital to every quarter of the globe in which they have practised the profession they had learnt within its walls.

| | |
|---|---|
| Samuel Gee | H. H. Tooth |
| Dyce Duckworth | A. E. Garrod |
| Philip Hensley | F. H. Champneys |
| Norman Moore | W. S. A. Griffith |
| John Langton | Anthony Bowlby |
| Howard Marsh | Charles B. Lockwood |
| W. J. Walsham | D'Arcy Power |
| Harrison Cripps | H. J. Waring |
| W. Bruce Clarke | W. McAdam Eccles |
| Samuel West | W. H. H. Jessop |
| J. A. Ormerod | W. T. Holmes Spicer |
| W. P. Herringham | A. E. Cumberbatch. |

*February*, 1903.

We have confidence that the Governors of St. Bartholomew's Hospital, the medical profession, and the public will be astonished at the temerity exhibited by those responsible for the suppression of the opinions of the Medical Council and the formulation of a scheme which practically fails to give effect to those opinions, or even to pay the staff the compliment of regarding them as material to the issue of any public appeal for funds on behalf of St. Bartholomew's Hospital.

### ST. BARTHOLOMEW'S HOSPITAL.

